# Thinner is not always better: Optimizing cryo-lamellae for subtomogram averaging

**DOI:** 10.1126/sciadv.adk6285

**Published:** 2024-04-26

**Authors:** Maarten W. Tuijtel, Sergio Cruz-León, Jan Philipp Kreysing, Sonja Welsch, Gerhard Hummer, Martin Beck, Beata Turoňová

**Affiliations:** ^1^ Department of Molecular Sociology, Max Planck Institute of Biophysics, Max-von-Laue-Straße 3, 60438 Frankfurt am Main, Germany.; ^2^ Department of Theoretical Biophysics, Max Planck Institute of Biophysics, Max-von-Laue-Straße 3, 60438 Frankfurt am Main, Germany.; ^3^ IMPRS on Cellular Biophysics, Max-von-Laue-Straße 3, 60438 Frankfurt am Main, Germany.; ^4^ Central Electron Microscopy Facility, Max Planck Institute of Biophysics, Max-von-Laue-Straße 3, 60438 Frankfurt am Main, Germany.; ^5^ Institute of Biophysics, Goethe University Frankfurt, 60438 Frankfurt am Main, Germany.; ^6^ Institute of Biochemistry, Goethe University Frankfurt, 60438 Frankfurt am Main, Germany.

## Abstract

Cryo–electron tomography (cryo-ET) is a powerful method to elucidate subcellular architecture and to structurally analyze biomolecules in situ by subtomogram averaging, yet data quality critically depends on specimen thickness. Cells that are too thick for transmission imaging can be thinned into lamellae by cryo–focused ion beam (cryo-FIB) milling. Despite being a crucial parameter directly affecting attainable resolution, optimal lamella thickness has not been systematically investigated nor the extent of structural damage caused by gallium ions used for FIB milling. We thus systematically determined how resolution is affected by these parameters. We find that ion-induced damage does not affect regions more than 30 nanometers from either lamella surface and that up to ~180-nanometer lamella thickness does not negatively affect resolution. This shows that there is no need to generate very thin lamellae and lamella thickness can be chosen such that it captures cellular features of interest, thereby opening cryo-ET also for studies of large complexes.

## Teaser

Lamellae can be up to 180 nm thick without losing resolution when performing cryo–electron tomography with subtomogram averaging.

## INTRODUCTION

Cryo–electron tomography (cryo-ET) enables three-dimensional (3D) observation of near-natively preserved cryogenically fixed biological specimens (*1*, *2*). When followed by subtomogram averaging (STA) (*3*, *4*), in situ cryo-ET combines high-resolution structural information of proteins or macromolecular complexes with contextual information within their functional crowded cellular environment (*5*, *6*). Apart from technical specifications of the imaging equipment, the quality of cryo-ET datasets is greatly influenced by the properties and quality of the sample. Because of the limited penetration power of electrons, sample thickness is a particularly major limitation (*7*). The influence of sample thickness on resolution is well established for single-particle cryo–electron microscopy (SPA cryo-EM) (*8*, *9*). Although many biological samples are thin enough to be imaged directly (*10*–*12*), other samples, most notably eukaryotic cells, need to be thinned prior to imaging (*13*). Currently, cryo–focused-ion beam milling (cryo-FIB milling) is the thinning method of choice, as it lacks several severe artifacts associated with cryo-sectioning (*14*, *15*). During cryo-FIB milling, material from the sample is ablated by focused ions, until a thin (up to ~300 nm) slice remains, which can subsequently be subjected to tomographic acquisition (*16*). The cryo-FIB to STA workflow has been successfully applied to a wide variety of samples and, recently, high-resolution maps have been obtained with local resolutions up to 2.4 to 3.5 Å (*17*, *18*). It is known that samples amass structural damage along their milling surface during cryo-FIB milling, induced by implanted ions or cascading secondary particles resulting from interactions with ions (*15*, *19*). In material sciences, this damage can be directly observed and has been quantified for many materials, ionic species, and ion energies (*20*–*23*). These results have previously been extrapolated to biological material (*15*) and recently experimentally tested for argon (*19*) and gallium (*24*) ions. The latter study used a 2D template matching (TM) approach (*25*) to assess damage; however, notably, no STA or averaging approaches were used to quantify the structural damage from cryo-FIB milling.

While the effect of cryo-ET dose distribution on the resolution attained by STA has previously been benchmarked (*26*), the effect of the use of lamellae and their properties on cryo-ET and STA has not yet been systematically investigated. Here, we benchmark the effect of the local lamella thickness on STA resolution and quantify the extent and degree of structural damage caused by gallium ions during the cryo-FIB milling process. By milling thinner lamellae, the contrast and signal-to-noise ratio (SNR) in the tilt images increases, likely resulting in better alignment of the tilt series before tomogram reconstruction, which is a crucial step that determines the quality of the tomogram and thereby all successive processing steps. Simultaneously, however, very thin lamellae are nontrivial to prepare and contain less of the cellular context, and larger particles may no longer completely fit inside the lamellae. Moreover, the volume that remains undamaged by ions is concomitantly smaller as well, resulting in a trade-off between contrast and SNR on one side and the ion damage layer on the other.

Here, we used ribosomes as a probe to determine the influence of lamella thickness on STA resolution and to assess the effect of ion-induced damage by gallium ions during cryo-FIB milling. We found that intermediate lamella thickness up to 180 nm and particles that are located at least 30 nm into the lamellae can be used without any loss of resolution. Overall, our systematic benchmark provides guidance for the milling of lamellae and the selection of particles to optimize resolution for STA.

## RESULTS

**Fig. 1.**
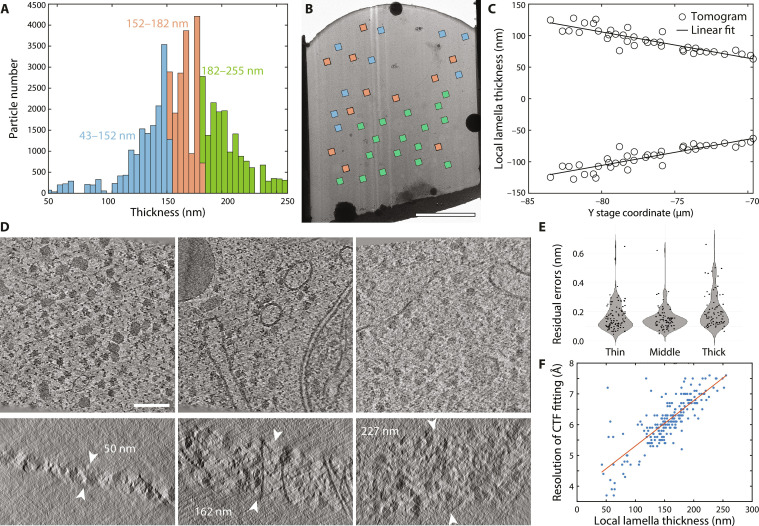
Cryo-tomography dataset overview. (**A**) Histogram of the local lamella thickness based on all subtomograms in the dataset. These were divided into three thickness groups, each containing 16,114 subtomograms. (**B**) Typical lamella imaged in a cryo–transmission electron microscope. Squares represent position and field of view of acquired tilt series, colored according to the local thickness scale shown in (A). (**C**) Projected local lamella thickness from front (right) to back (left) of the lamella shows the wedge shape due to cryo-FIB geometry. Local thickness was divided by 2 and mirrored toward negative Y scale for visualization. (**D**) *XY* (top) and *XZ* slices (bottom) through reconstructed tomograms from each thickness group, displaying local thickness of the lamella between the white arrowheads. (**E**) Violin plot of the residual errors after tilt-series alignment for each thickness group. (**F**) Resolution up to which CTF was reliably fitted in Warp for every tilt series in the dataset plotted against the local thickness of the lamella. The red line represents a linear fit through all data points. Scale bars, 5 μm (B) and 100 nm (D) (applies to all subpanels).

For this study, we extended a previously collected cryo-ET dataset of *Dictyostelium discoideum* cells (*17*) to a total of 261 tilt series collected from 12 lamellae, by acquiring additional tilt series on a Titan Krios G4 equipped with a cold field emission gun, Selectris X energy filter (set to a slit width of 10 eV), and a Falcon 4 camera, at a pixel size of 1.2 Å, using a total dose of 120 *e*^−^/Å^2^ (also see Materials and Methods). After preprocessing and tomogram reconstruction in IMOD (*27*) (see Materials and Methods), the thickness of the lamella within each tomogram was measured manually, which will be referred to as the local lamella thickness. We found that local lamella thickness ranged from 43 to 255 nm (see Fig. 1A). Due to the geometry of the ion beam during the cryo-FIB milling process, local lamella thickness generally increases from the front toward the back of the lamella, creating a wedge shape (see Fig. 1, B and C, and fig. S1) (*28*). Tomograms were reconstructed again using Warp (*29*), and ribosome particles were identified by TM in STOPGAP (*30*). We extracted ~168,000 particles as putative ribosomes, which are hereafter referred to as the raw template matches. To filter out false positives, i.e., particles other than ribosomes, we subjected the raw template matches to multiple rounds of 3D classification on binned data in Relion 3.1 (*31*), after which ~48,000 ribosomal particles were retained for further analysis (see figs. S2 and S3).

### Averaging ribosomes from lamellae with local thickness up to ~180 nm has no adverse effect on resolution

To study the effect of local lamella thickness on the resolution obtained by STA, we divided all subtomograms of ribosomes in equally sized groups, based on the local lamella thickness. This resulted in the three thickness groups: 43 to 152 nm (“thin”), 152 to 182 nm (“middle”), and 182 to 255 nm (“thick”) (see Fig. 1, A and D, and figs. S4 and S5), each containing 16,114 particles. Beyond cryo-FIB damage and reduced electron penetration power due to increased sample thickness, tilt series alignment is of critical importance to the quality of the tomograms (*32*). Cryo-FIB–milled lamellae lack high-contrast fiducial markers, and the contrast is further reduced by the crowded cellular interior, which is exacerbated for thicker lamellae (see fig. S6). Unexpectedly, we found that the residual errors after tilt series alignment only vary slightly for the three thickness groups (see Fig. 1E). Another important parameter to consider is the fitting of the contrast transfer function (CTF) on the tilt images; the precision of which is dependent on the thickness of the sample. Warp estimates the resolution up to which the CTF can be reliably fitted in 2D images (*29*), and we found a linear dependency on this estimated resolution against the local thickness (see Fig. 1F). Note that for lamellae of thicknesses discussed here, this does not limit the resolution that can be achieved by STA but rather reflects the strength of the frequency signal in the images.

**Fig. 2.**
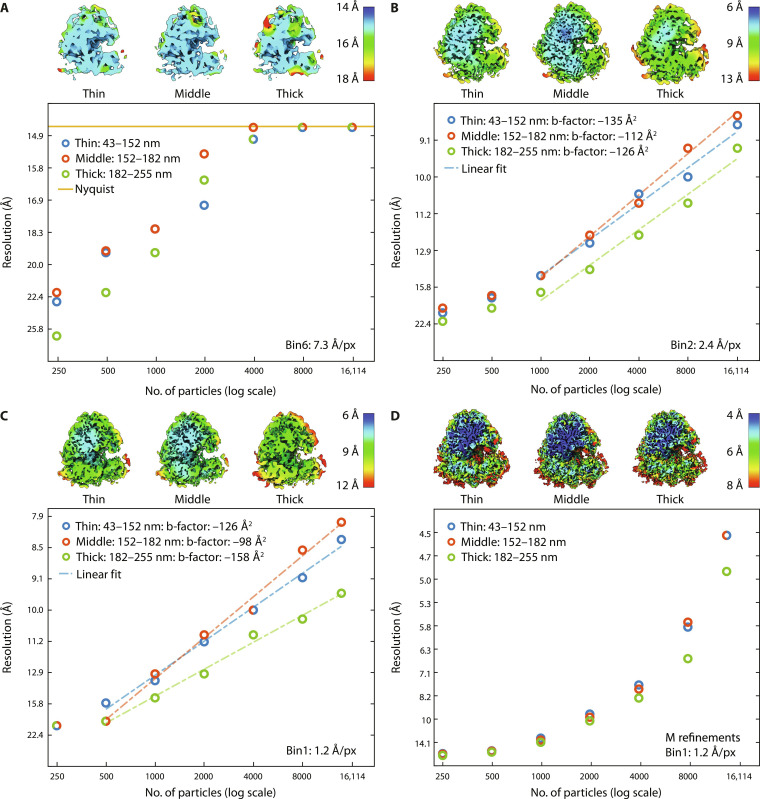
Local resolution density maps and resolution plots of different binning factors for lamellae with various local thicknesses. Resolution is plotted on the vertical axes (on square inverse scale). The number of particles on horizontal axes is on log scale. Top: Density maps of the largest particle subset for each thickness group. Bottom: Resolution plotted against randomly selected subsets of particles. (**A**) The results for subtomograms extracted with a binning factor of 6. (**B**) Results for a binning factor of 2, with linear fit through the linear regime of the data (*33*). (**C**) Results for unbinned data, processed with Relion 3D refinement only. (**D**) Results for unbinned data further processed with M.

All ribosomal particles were extracted using Warp (*29*) and subjected to 3D refinements in Relion 3.1. No distinction was made between particles close to the lamella surface or deeper within the lamellae. To quantify the quality of data coming from each of the thickness groups, we analyzed the respective particle subsets by plotting the square inverse resolution versus particle numbers [a so-called Rosenthal-Henderson plot (*33*); see Fig. 2]. This procedure was performed on particles extracted with binning factors of 6 and 2, as well as unbinned (bin1), to determine the pixel size at which lamella thickness would potentially start to play a role. First, particles were extracted with a binning factor of 6 (corresponding to a pixel size of 7.3 Å), which reached the Nyquist limit when more than 4000 particles were used, regardless of lamella thickness (see Fig. 2A). Thus, for lower magnification cryo-ET and STA, lamella thickness up to 255 nm does not negatively influence the resolution. Similar analysis with a binning factor of 2 and unbinned (corresponding to pixel sizes of 2.4 and 1.2 Å, respectively) clearly separated particles in the middle and thinner thickness groups that converged to higher resolution from those extracted from thick lamellae (Fig. 2, B and C). The particles from the middle thickness group slightly outperformed those from the thinnest lamellae. We speculate that the mechanical stability of medium thickness lamellae versus thin lamellae during imaging could be a factor that affects the resolution.We finally refined the unbinned (bin1) particles in M (*34*), which is known to further improve the resolution by local refinement (*10*, *17*, *18*). Indeed, we found a notable increase in resolution for each of the particle groups (see Fig. 2, C and D). Both thin and middle thickness groups converged to the same resolution (4.5 Å), whereas the particles from the thicker lamellae converged to 4.9 Å. We conclude that lamellae with a local thickness up to ~180 nm can be used without compromising the resolution. Thinner lamellae are not necessarily required for achieving the highest resolution.

### Cryo-FIB milling causes structural damage to the sample up to ~30 nm from the surface

**Fig. 3.**
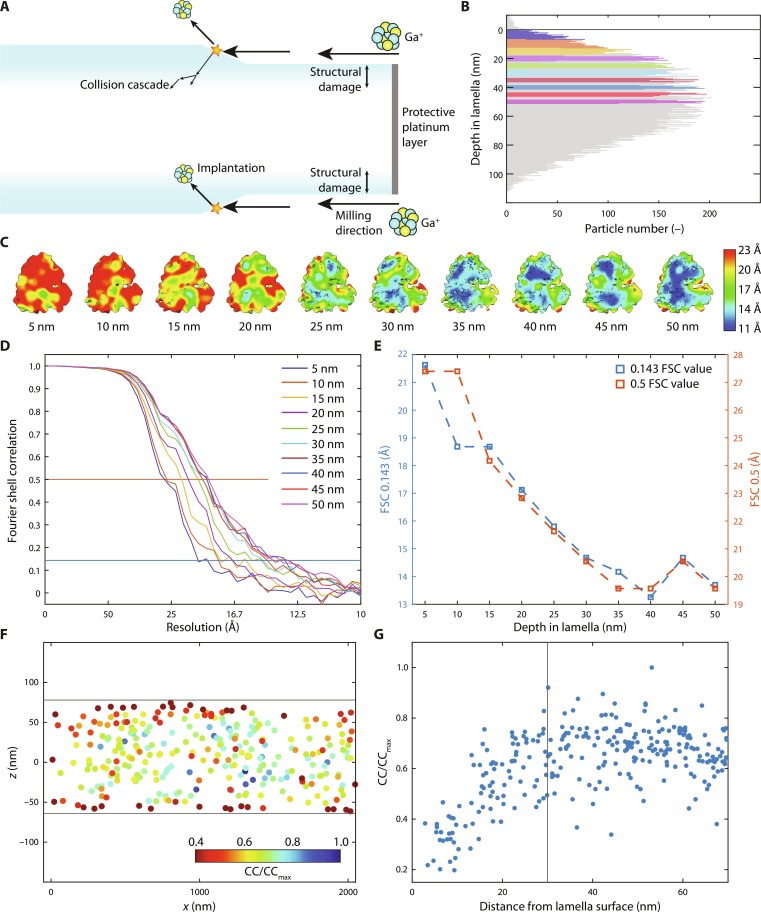
Ion-induced structural damage inside lamellae. (**A**) Milling geometry inside the cryo-FIB microscope. Upon milling and removing material, ions are implanted, and cascade events cause structural damage in the lamella. (**B**) Histogram of all particles in the analysis, plotted with respect to the closest lamella surface (gray line). Colors indicate particles extracted for analyses for each of the depth groups. (**C**) Subtomogram averages of 1000 particles from each depth, colored according to local resolution. (**D**) Fourier shell correlation (FSC) of each depth group, using the colors from (B). (**E**) Plot of the FSC value at cutoff values 0.5 and 0.143 [shown as horizontal lines in (D)] for each particle subgroup. (**F**) CC scores, normalized by their maximum value (CC/CC_max_) from 3D high-resolution TM. Lamella surfaces are displayed as horizontal lines. (**G**) Normalized CC scores (CC/CC_max_) plotted versus the distance from the lamella surface, showing the same data as shown in (F). Vertical line is the 30-nm ion damage zone that was found using the STA approach shown in (C) to (E).

We then sought to quantify the depth and extent of structural damage caused by implanted ions and the collision cascade originating from cryo-FIB milling (see Fig. 3A). To control for the influence of thickness described above, we restricted the local lamella thickness to 140 to 190 nm for this analysis. To accurately extract particles at a given depth from the lamella surface, we introduced additional preprocessing steps that take into account the geometry of the sample (see Materials and Methods and fig. S7 for details). To quantify ion damage in particles located close to the lamella surface, we selected particles from depths of 5 to 50 nm and compiled them in groups of 5-nm intervals, with 1000 particles in each group (see Fig. 3B). These were then extracted at a binning factor of 2 (pixel size of 2.3 Å) and refined using Relion 3.1. We found that particles centered around 5 nm from the surface averaged to 21.6-Å resolution. When particles were extracted from deeper within the lamellae, the resolution improved steadily until a plateau was reached at a depth of roughly 30 nm, where particles could be resolved up to 14.7- to 13.3-Å resolution (see Fig. 3, D and E). Particles in the 5- to 15-nm distance range from the lamella surface likely constitute of ribosomes that are partly ablated during cryo-FIB milling, whereas particles from the 15- to 30-nm distance range are more likely the result of implantation, collision cascades, or other secondary sources.

An alternative approach to quantifying the depth of the ion damage zone is to perform TM with a high-resolution template and to analyze the cross-correlation (CC) scores of the particles as a proxy for damage (*35*). By performing TM on tomograms constructed with a binning factor of 2 (corresponding to a voxel size of 2.4 Å), we intended to include higher frequencies (up to ~5 Å) in our analysis. We found that particles from close the lamella surface showed reduced CC scores compared to particles found deeper within the lamellae (see Fig. 3, F and G, and fig. S8). CC scores from particles found deeper than ~30 nm leveled off to a constant regime, agreeing well with the STA results presented above.

We then wondered whether the ion damage layer could account for the small disparity between the thin and middle groups, shown in Fig. 1C, as the particles from the thin group may be proportionally more affected by the ion damage. We therefore selected only particles further than 30 nm away from the lamella surface from both thin and middle thickness groups (equalizing particle numbers) and refined these in Relion as before. We found that the particles from the middle thickness group still averaged to slightly higher resolution (8.0 and 8.3 Å for middle and thin, respectively; see fig. S9) in agreement with the existing trend. Hence, the small disparity between middle and thick cannot be explained by the ion damage. These data clearly show that particles extracted closer to ~30 nm from either surface of the lamellae are of lower quality than those deeper inside the lamellae.

### Resolution of ribosomes slightly decreases from the front toward the back of the lamellae

**Fig. 4.**
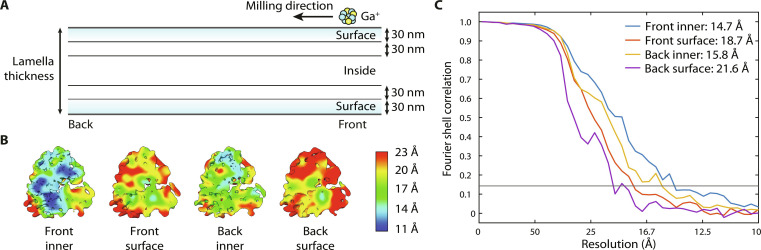
Resolution comparison between front and back of the lamella. (**A**) Milling geometry with respect to the front (left side) and back (right side) of the lamella. Particles from the first 30 nm (top and bottom) were compared to particles much deeper inside the lamella (>60 nm from the surface). (**B**) Subtomogram averages of each of these particle groups, colored according to local resolution. (**C**) FSC plot of the particle groups in (B).

As the cryo-FIB milling process is directed from the front toward the back of the lamellae, we analyzed a potential quality difference of particles originating from specific regions. Again, to avoid any thickness effects, we limited the local thickness to 145 to 189 nm for this study. The data were split into particles from the damage layer (<30 nm from the surface, “surface”) and particles well away from damaged regions of the lamellae (>60 nm from the surface, “inner”) (see Fig. 4A). This led to four particle groups (front inner, front surface, back inner, and back surface) of ~600 particles each, which were extracted and refined at a binning factor of 2 as before. While we detected a slight difference between particles from the front versus the back, both from inside (14.7 Å versus 15.8 Å, respectively) and from the surface of the lamellae (18.7 Å versus 21.6 Å, respectively) (see Fig. 4, B and C), the quality of the data is dominated by the damage layer.

### High-quality particles average to higher resolution

**Fig. 5.**
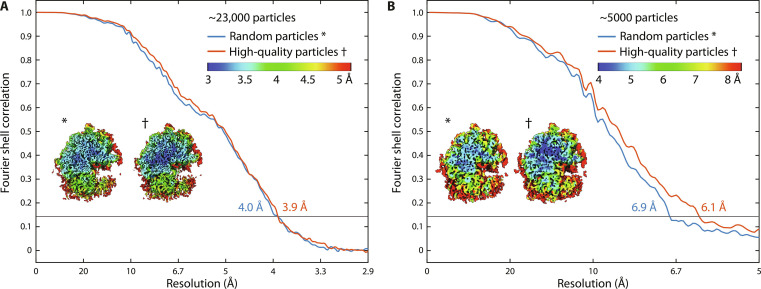
STA with high-quality particles compared to random particles. (**A**) FSC curve of 22,586 high-quality particles (red line) compared to 22,586 randomly selected particles (blue line). Inset: Local resolution map of both sets. (**B**) FSC curve of 4795 high-quality particles (red line) compared to 4795 randomly selected particles (blue line). Inset: Local resolution map of both sets.

As mentioned above, we found that particles originating from tomograms with local thickness below 180 nm and particles further away than 30 nm from each lamella surface averaged to higher resolution. We selected these high-quality particles from the entire dataset, which resulted in ~23,000 particles. To our surprise, these high-quality particles reached a similar resolution to an identical number of particles that were not filtered according to these quality criteria, 3.9 and 4.0 Å, respectively (see Fig. 5A). We hypothesized that although the randomly selected particles also contained particles of lower quality, this was most likely compensated by the relatively large total number of particles. Therefore, we also isolated two sets of ~5000 particles, high-quality and randomly selected from the whole dataset. The high-quality particle set averaged to 6.1-Å resolution, while the randomly selected particles averaged to 6.9 Å (see Fig. 5B). Thus, using high-quality particles results in higher resolution. However, the reduced quality of particles close to the surface or from thicker lamellae can be compensated by increasing the number of particles.

## DISCUSSION

Cryo-FIB milling of thick biological samples is gaining importance as in situ tomography of macromolecular complexes in (eukaryotic) cells becomes more widely used. Since its development for biological specimens and despite the recent surge in studies using this technique, no systematic assessment of its impact on structural output is available to date. In this work, we used ribosomes to benchmark the effect of cryo-FIB lamella preparation on resolution attained by STA. Notably, for the applied total dose of 120 *e*^−^/Å^2^, we found that particles from thinner (up to 150 nm) lamellae did not reach higher resolution than from intermediate thickness (up to 180 nm). As our studies only included data from *D. discoideum* cells, we cannot exclude that cell-specific characteristics, such as molecular crowding, could affect these results. Previously, the effect of recent technological advances on the resolution of SPA cryo-EM has been investigated (*9*), showing that 300-kV acceleration voltage, energy filters, and super-resolution detection strategies are particularly effective in thicker ice, all of which were exploited in the present study. Cryo-ET may benefit even more strongly from these technological advances than SPA, for example, by enabling higher-accuracy tilt-series alignment, thereby greatly reducing the thickness effects from the lamellae on resolution. Our finding that slightly thicker lamellae can be used without compromise on the attainable resolution is of great practical use, as the milling of very thin lamellae is not straightforward because of many technical challenges. Even thicker lamellae can be comfortably used if high resolution is not the main focus.

In addition to the contribution of lamella thickness, we also assessed the effect of ion damage during cryo-FIB milling on resolution and found that it causes structural damage to the sample up to ~30 nm from each surface. Particles from deeper layers in the lamella all averaged to roughly the same resolution. In addition, we confirmed these results by performing 3D TM and found that CC scores increased from the lamella surface up to ~30 nm and then remained constant. These results agree well with the damage layer found when using argon ions in a plasma FIB (*19*) but may differ for different ionic species, such as xenon, nitrogen, or oxygen. Heavier particles, such as xenon ions have a higher milling rate with concomitant shorter milling times, which resulted in a reduction in ion damage in silicon samples (*36*). However, we note that the detected depth of 30 nm is comparable to the size of our probe, the ribosome, and that particles that are closer to the surface are physically cut off by the ion beam. Therefore, our estimate for the depth of damage by implantation constitutes a maximum. We cannot exclude that the damaged layer is actually smaller. A recent study that used 2D TM to probe the CC score between the sample and a high-resolution template reported a reduction of the SNR for ribosomes up to ~60 nm from the lamella surface (*24*). However, this study did not perform any STA and instead used 2D images collected at high dose corresponding to SPA image acquisition parameters. Here, we used 3D TM, thereby including critical steps of tilt alignment and 3D CTF correction in the analysis.

Possible strategies to reduce ion damage in lamellae include using lower ion voltages or ion acceleration voltage for the final polishing step, as was suggested by a recent study (*37*), and strictly reducing the time that milling patterns remain activated near the lamella during polishing. The effect these strategies may have on the depth and severity of the ion damage layer needs to be investigated in future work.

When a relatively small number of particles were averaged, high-quality particles from lamellae with local thickness of less than 180 nm and located deeper than 30 nm in the lamella averaged to a higher resolution. However, our dataset of ~23,000 particles and other previously published high-resolution datasets (*17*, *18*) suggest that the thickness effect and ion damage are not a major concern for the cryo-FIB milling and cryo-ET workflow and can be compensated by including more particles in the average.

We note, however, that the ion damage is only apparent when performing STA or TM. Visually, all tomograms appeared undamaged, unlike for, e.g., crystalline material (*38*) and cryo-sectioning techniques where the ion damage or structural deformation is clear from visual inspection (*39*). Thus, for morphological studies, the ion damage does not have to be considered.

Together, our systematic analysis shows the effect of cryo-FIB milling on the resolution achieved by STA. We found a minor loss of quality from particles originating from thicker parts of lamellae or from close to the lamella surface, resulting in a decrease in resolution. However, for many cases, such as lower-resolution averaging and morphological studies, these effects are negligible, and the data can be used without concern regarding ion damage. Even for high-resolution studies, we have shown that, despite the presence of lower-quality particles, these effects can be countered by expanding the dataset to include more particles. These results show that cryo-FIB milling in general is not impeding any high-resolution structural studies.

## MATERIALS AND METHODS

### Cryo-EM sample preparation

*D. discoideum* strain Ax2-214 cells were grown in HL5 medium (Formedium) containing ampicillin (50 μg/ml) and geneticin G418 (20 μg/ml; Sigma-Aldrich) at ~20°C. Au grids (200 mesh) with R1/4 SiO_2_ foil (Quantifoil) were glow-discharged for 90 s at 0.38 mbar and 15 mA using a Pelco easiGlow device. Exponentially growing cells were diluted to a concentration of 3.3 × 10^5^ cells/ml. A droplet of 100 μl of cell suspension was placed on the glow-discharged grids, and cells were allowed to attach to the grid for 2 to 4 hours at room temperature. Cells were subsequently vitrified by plunge-freezing into liquid ethane using a Leica GP2 plunger. Lamellae were prepared by cryo-FIB milling with an Aquilos FIB–scanning electron microscopy (SEM) (Thermo Fisher Scientific). Before milling, grids were coated with organometallic platinum layer using a gas injection system for 10 s and additionally sputter-coated with platinum at 1 kV and 10-mA current for 20 s. SEM imaging was performed with 10 kV and 13-pA current to guide the milling progress. Rough milling was performed using SerialFIB (*40*) in three steps. Distance between milling patterns were reduced from 3 to 1.2 μm and 600 nm, using 500-, 300-, and 100-pA current for 300, 300, and 200 s, respectively. Fine milling was performed manually at 30 pA, starting with the patterns 250 nm apart. The top pattern was kept at the same position, while the bottom pattern was raised in small steps to make the lamella thinner. All steps were carried out with 30-kV acceleration voltage. During polishing, intermittent SEM scans were used to ensure that the protective platinum layer was still intact. Lamellae were targeted to a thickness between 75 and 180 nm. Roughly half of the lamellae were sputter-coated with platinum after polishing for 1 s at 1 kV and 10 mA.

### Cryo-ET data acquisition

The cryo-ET dataset was acquired in two microscope sessions, from 12 lamellae on two grids. Data were collected on a Titan Krios G4 microscope operated at 300 kV equipped with a cold field emission gun, Selectris X imaging filter, and Falcon 4 direct electron detector, operated in counting mode (all Thermo Fisher Scientific). Overview montages of individual lamellae were acquired with 3.0-nm pixel size to select suitable areas for tilt-series collection. Tilt series were acquired using SerialEM (version 4.0.1) in low-dose mode as 4096 × 4096 movies of 10 frames each and on-the-fly motion–corrected in SerialEM. The magnification for projection images of 105,000 × corresponded to a pixel size of 1.223 Å. Tilt-series acquisition started from the lamella pretilt of +8° and a dose symmetric acquisition scheme (*41*) with 2° increments grouped by 2 was used, resulting in 61 projections per tilt series with a constant exposure time and targeted total dose of ~120 *e*^−^/Å^2^. An energy slit with 10-eV width was inserted, and the nominal defocus was varied between −2.5 and −4.5 μm. Dose rate on the detector was targeted to be ~6 *e*^−^ per pixel per second at the untilted specimen on a representative position on a lamella.

### Image processing, TM, and classification

The motion corrected tilt series were dose-filtered using MATLAB-based scripts (*42*) and cleaned by visual inspection. The dose-filtered tilt series were then aligned through patch tracking and reconstructed as back-projected tomograms with SIRT-like filtering of 10 iterations at a binned pixel size of 4.9 Å [both done in IMOD 4.11.5 (*27*)]. From the reconstructed tomograms 216 were selected by visual inspection. Tilt series were rejected on the basis of the tilt images being very blurry, image acquisition was aborted for experimental reasons, or the reconstructed tomogram was extremely blurry. For compatibility with Relion (*31*) and M (*34*), the selected tilt series were then reprocessed in Warp (*29*) with the alignment obtained from IMOD (see fig. S2 for a complete overview).

For TM, a ribosome average was generated using Relion from approximately 1000 manually picked ribosomes, with a binning factor of 10 (12.2 Å per pixel). TM was performed with the initial ribosome average on deconvolved tomograms with a binning factor of 10 reconstructed in Warp using STOPGAP (*30*). For each tilt series, the 800 highest CC peaks were selected; their coordinates exported and converted to a Warp-compatible star-file using the dynamo2m toolbox (*43*).

The 168,000 positions determined through TM were extracted as subtomograms in Warp at a binning factor of 6 (7.338 Å per pixel) and subjected to multiple rounds of 3D classification using Relion 3.1 to filter out junk particles (see fig. S3). This yielded 48,342 ribosomal particles, which were used for successive processing steps (also see figs. S2 and S3).

### Particle curation and refinement for the thickness analysis

For the thickness analysis, the thickness of the lamellae was measured manually using IMOD. Three thickness groups were created by equalizing the number of particles among these groups. This resulted in thickness ranges of 43 to 152 nm, 152 to 182 nm, and 182 to 255 nm, each containing 16,114 particles (see fig. S5). Lists of particle indices referring to the particle starfile exported from STOPGAP, cross-referenced to the classification results, were created in MATLAB, which served as the input for starparser (*44*) to create separate starfiles with particles for each thickness group. Separate Warp projects were made for each of the thickness groups. To create Rosenthal-Henderson plots, subsets of particles (of 250, 500, 1000, 2000, 4000, 8000, and 16,114 particles) were created by randomly reducing the number of particles in the starfile in a sequential manner, using starparser. The random selection of particles was checked to ensure unbiased thickness distribution in the subsets. For the unbinned data instead of 16,114 particles, only 13,840 particles could be extracted because of computational limits; however, particle numbers between the thickness groups were kept identical.

Then subtomograms with a binning factor of 6 (corresponding to a pixel size of 7.3 Å per pixel) and 2 (2.4 Å per pixel) and unbinned subtomograms (1.2 Å per pixel) were extracted using Warp (downsampling or binning was performed in Fourier space) and refined and postprocessed in Relion 3.1. Refinement results from previous binning factors were used to reextract subtomograms at lower binning to have better initial particle orientation and speed up further refinement runs. Next, on the basis of the refinement of unbinned subtomograms in Relion, successive refinements were carried out in M (version 1.0.9). Multiparticle refinement of the tilt-series, geometric, and CTF parameters were refined in a sequential manner until the resolution no longer improved (see table S1). Care was taken to first process the 250-particle subset and then continue with the larger subsets to avoid influence of the multiparticle alignments from a refinement with higher particle numbers. Linear fits for the Rosenthal-Henderson plot were created in MATLAB. Note that for the M-refined data, no linear regime was found to fit for a b-factor, due to M’s multiparticle refinement, and because of the way the random selection of subsets was selected, the number of particles per tomogram increased, thus increasing the resolution in a nonlinear manner.

### In silico straightening of ribosome coordinates for ion damage analysis

To avoid thickness effects, only tomograms containing lamellae with local thickness in the range of 140 to 190 nm were selected. First, the distance from each particle to the lamella surface had to be accurately determined. For various reasons, such as pretilt, offset between loading from the cryo-FIB microscope to the transmission microscope and inherent nonflatness of the lamellae, the cellular material is not aligned with the *XY* plane in the reconstructed tomograms. Therefore, the ribosome coordinates were straightened within each tomogram, and the edges of the lamellae were determined. Using custom MATLAB scripts, a plane was fitted through the raw ribosome template coordinates before classification (see fig. S7A). For each raw ribosome coordinate, the *Z* coordinate of the fitted plane was subtracted from the *Z* coordinate from TM, which resulted in a flattened lamella, roughly centered around *Z* = 0 (see fig. S7B). From a histogram of all *Z* coordinates from one tomogram, the positions of both the top and bottom lamella edge were estimated as where the number of template matches drops off sharply around the edges of the lamella (see fig. S7, B and C). Then, the distance from the ribosome coordinate to the closest lamella surface was calculated, where no distinction between top and bottom surfaces was made. This script has been made available in cryoCAT (https://github.com/turonova/cryoCAT).

Particles originating from specific depths in the lamellae were selected as follows. A total of 1000 particles most closely centered around 5 nm in depth from the surface were first selected. This was then repeated with 5-nm intervals until 50 nm from the lamella surface, and for each depth, 1000 closest particles were selected, using a custom MATLAB script and used as input for starparser. For the 5- and 10-nm distance group, there is an overlap in particles, as not enough particles were located so close to the surface of the lamella.

Subtomograms were extracted with a binning factor of 2 using Warp, corresponding to a pixel size of 2.4 Å per pixel. These subtomograms were refined and postprocessed with a solvent mask in Relion for accurate Fourier shell correlation (FSC) comparison. Last, local resolution jobs were run in Relion.

For the analysis of the resolution between the front and back of the lamella, tomograms from the front and back were selected manually, chosen such that the thickness range was 145 to 189 nm for all. Then, particles were grouped into close to the surface (within 30 nm from the lamella surface) or particles that were >60 nm away from the surface to avoid any ion damage. These four groups were processed like before, extracted with a binning factor of 2 using Warp and refinement, and postprocessing and local resolution jobs were run in Relion.

### High-confidence TM for ion damage analysis

High-confidence 3D TM was performed using GAPSTOP (https://gitlab.mpcdf.mpg.de/bturo/gapstop_tm) as previously described (*35*). To include higher-frequency information, TM was performed on four tomograms with a binning factor of 2, corresponding to a pixel size of 2.4 Å, using angular steps of 5°, which resulted in 119,952 rotations. The template used was the high-resolution 80*S* ribosome (EMD-15807) derived from the same dataset (*17*), filtered with a low-pass filter of 5 Å. Peaks greater than 20 SDs above the background noise were extracted and correlated with the peaks used in this study from the same tomograms to validate the peaks. CC scores were normalized to the maximum CC score for each tomogram and used as metric CC/CC_max_.

### High-quality particle selection and processing

To select high-quality particles from the entire dataset, first, tomograms with local thickness smaller than 180 nm were selected. Then, particles from the damage zone (closer than 30 nm from the lamella surface) were excluded. These particles were extracted in Warp, refined in Relion, and successively refined in M, as described above. As comparison, tomograms and particles were randomly selected so that the number of particles were identical to the high-quality dataset, and these were processed in an identical fashion.

Then, for the smaller subset of high-quality particles, tomograms were randomly selected until 4795 high-quality particles could be extracted. These were compared with particles from randomly selected tomograms until 4795 particles could be selected. By selecting tomograms rather than particles, the effect of the multiparticle approach in M refinement was maximized and also mimicked realistic datasets. Again, particles were processed in Relion and M similar as before. All density maps and local resolution maps were plotted using UCSF ChimeraX (*45*).

## Supplementary Materials

### This PDF file includes:

Figs. S1 to S9 Table S1 References
