# Supplementary material for: Thinner is not always better: Optimizing cryo-lamellae for subtomogram averaging
Source: Sci Adv. 2024 Apr 26;10(17):eadk6285. doi: 10.1126/sciadv.adk6285 (PMC11051657; doi:10.1126/sciadv.adk6285)
Supplement: Supplementary file 1 — Figs. S1 to S9 Table S1 References [file sciadv.adk6285_sm.pdf]

Science Advances
AAAS

Supplementary Materials for

Thinner is not always better: Optimizing cryo-lamellae for subtomogram averaging

Maarten W. Tuijtel *et al.*

Corresponding author: Gerhard Hummer, gerhard.hummer@biophys.mpg.de; Martin Beck, martin.beck@biophys.mpg.de; beata.turonova@biophys.mpg.de

Sci. Adv. **10**, eadk6285 (2024)
DOI: 10.1126/sciadv.adk6285

This PDF file includes:

Figs. S1 to S9
Table S1
References

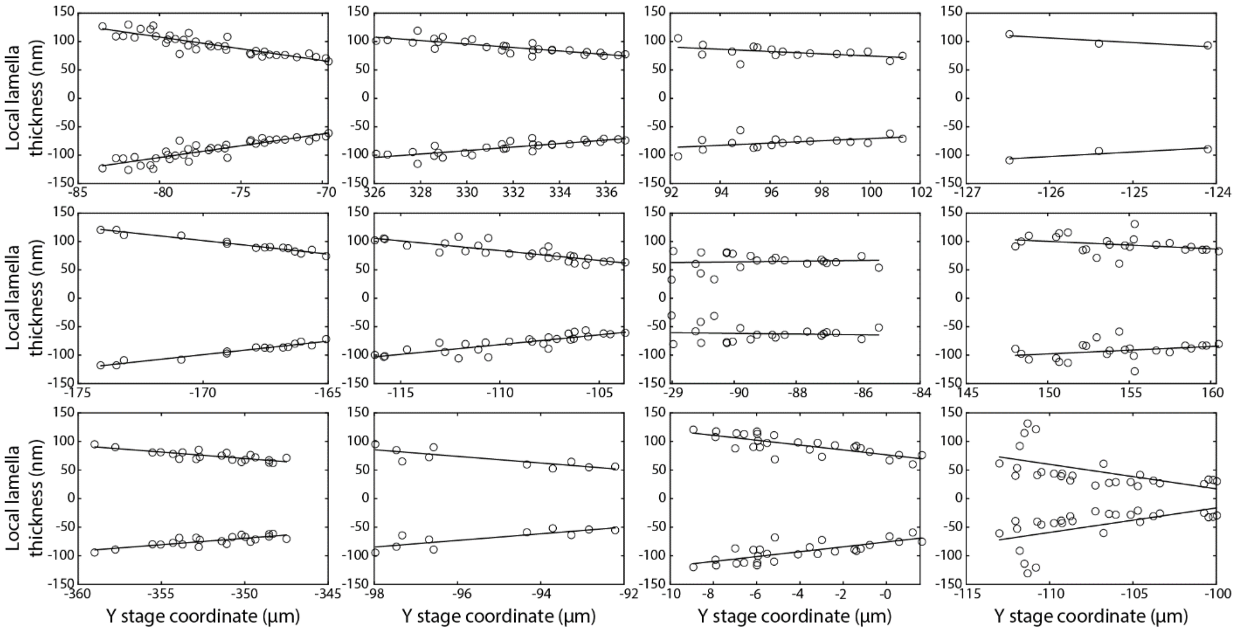

Fig. S1. Projected local lamella thickness profiles of all lamellae used in this study. Local lamella thickness of each tomogram was divided by two, mirrored towards negative Y-scale and plotted versus the Y stage coordinate during tilt-series acquisition. Circles represent acquired tilt-series and lines are linear fits through the points. Milling direction in the cryo-FIB was from right to left. The lamella shown in the bottom-right panel consisted of two cells, which could only be milled in two parts with relatively large spread in local thickness, thereby reducing the accuracy of the single linear fit.

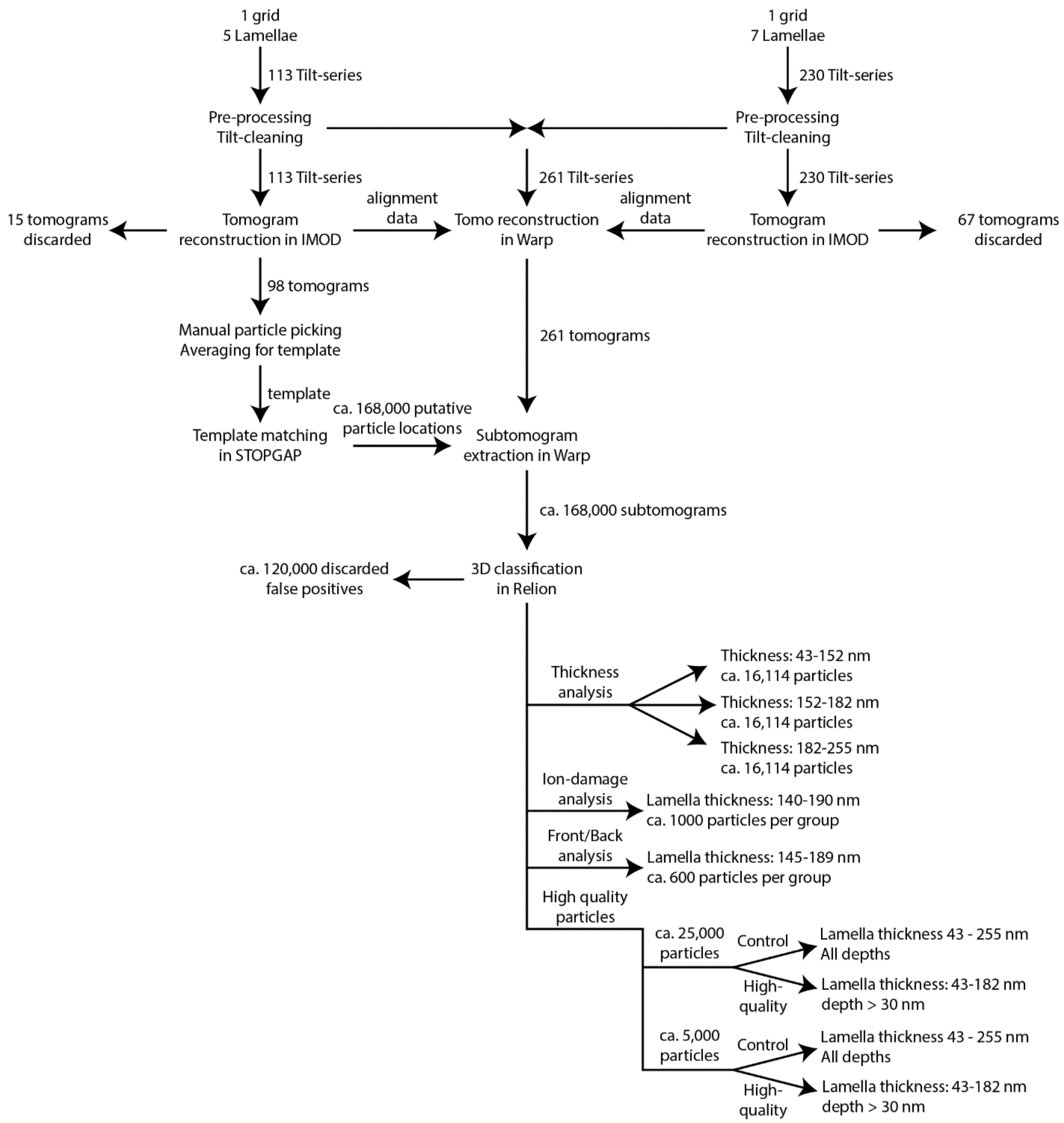

Fig. S2. Processing workflow

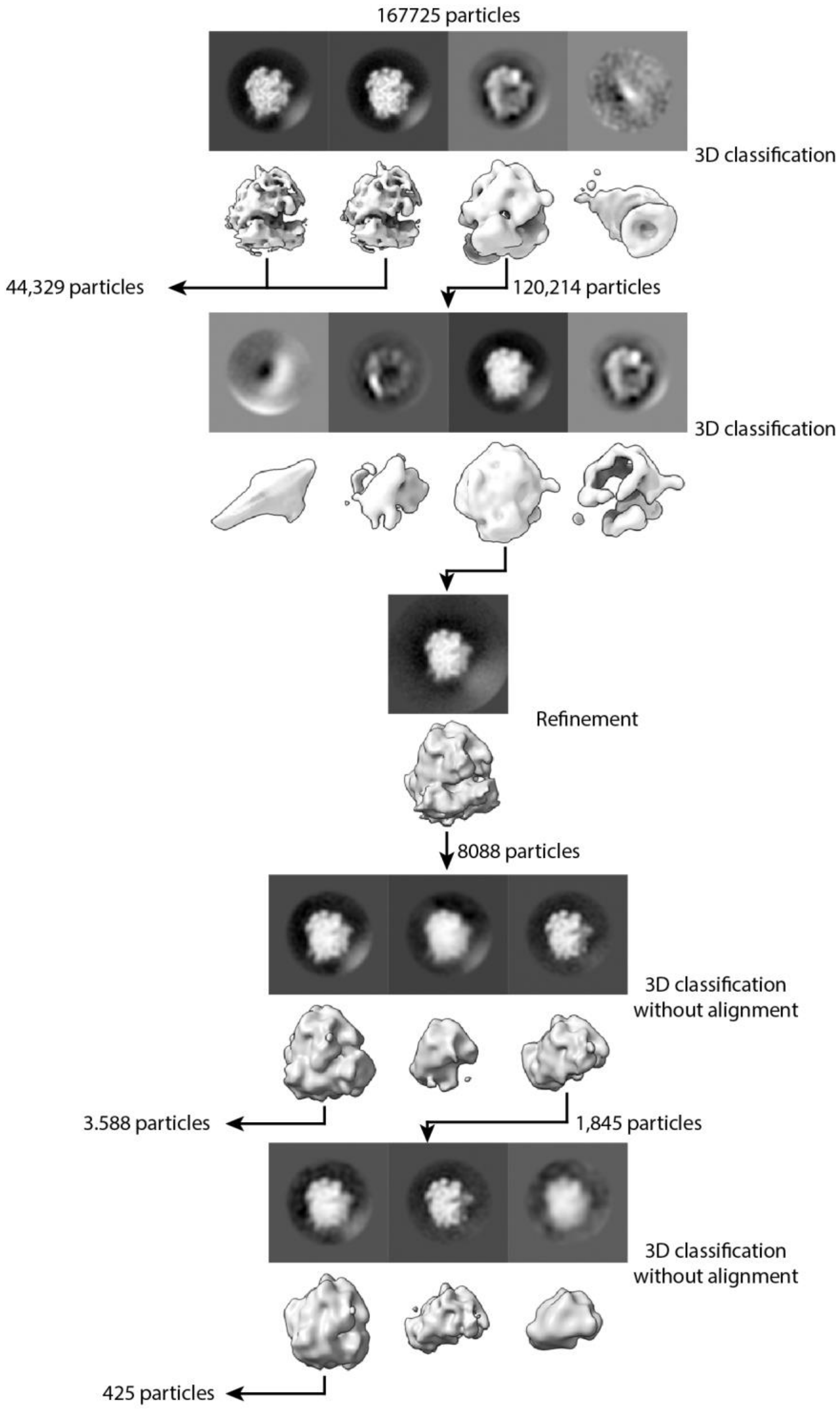

Fig. S3. Details of the 3D classification routine to exclude false-positive template matches.

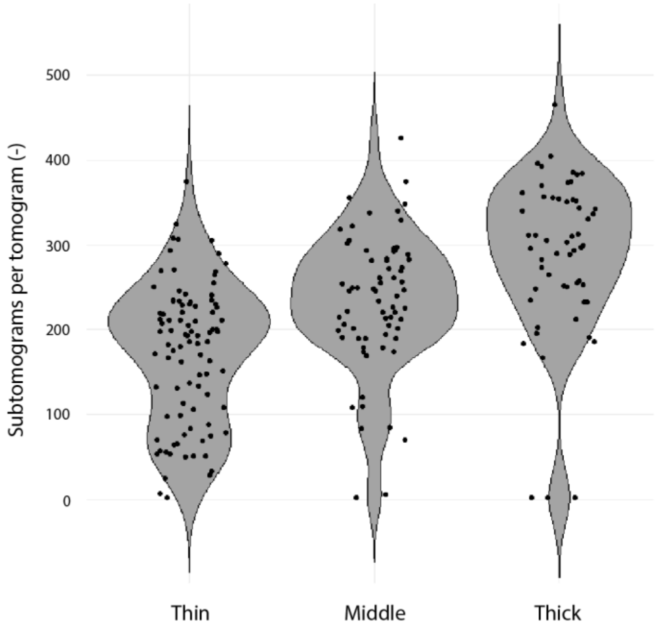

Fig. S4. Number of subtomograms per tomogram for each thickness group.

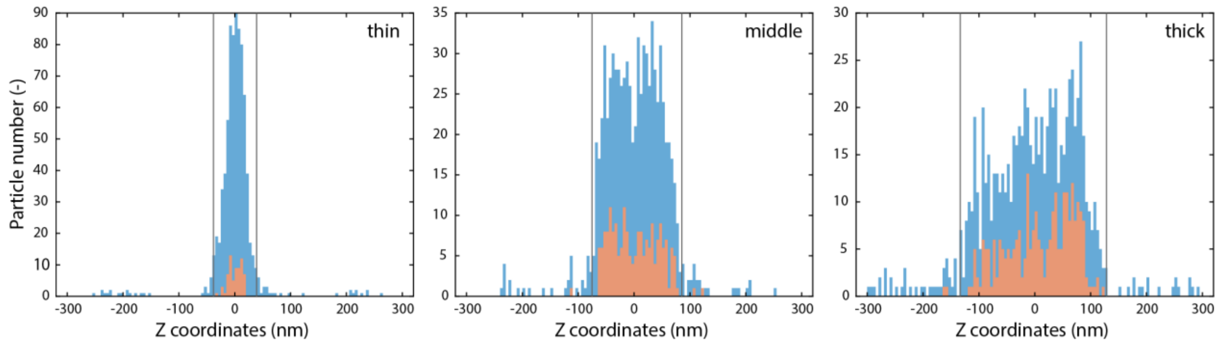

Fig. S5. Histogram of z-coordinates of the raw template matches (blue) and ribosomal particles (red). The tomograms correspond to the example tomograms shown in Fig. 1D. Lamella thickness is indicated by the vertical lines.

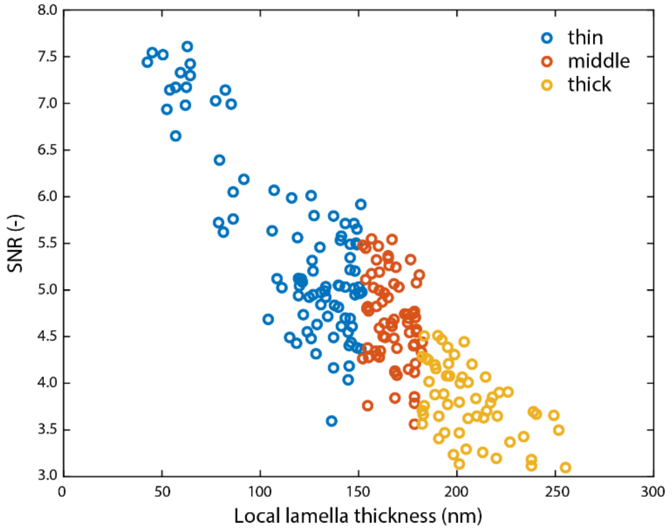

Fig. S6. SNR for all tilt-series used in this study. SNR was calculated by dividing the square of mean by the square of the standard deviation for each tilt-image at zero tilt, or pre-tilt of the lamella (*46*).

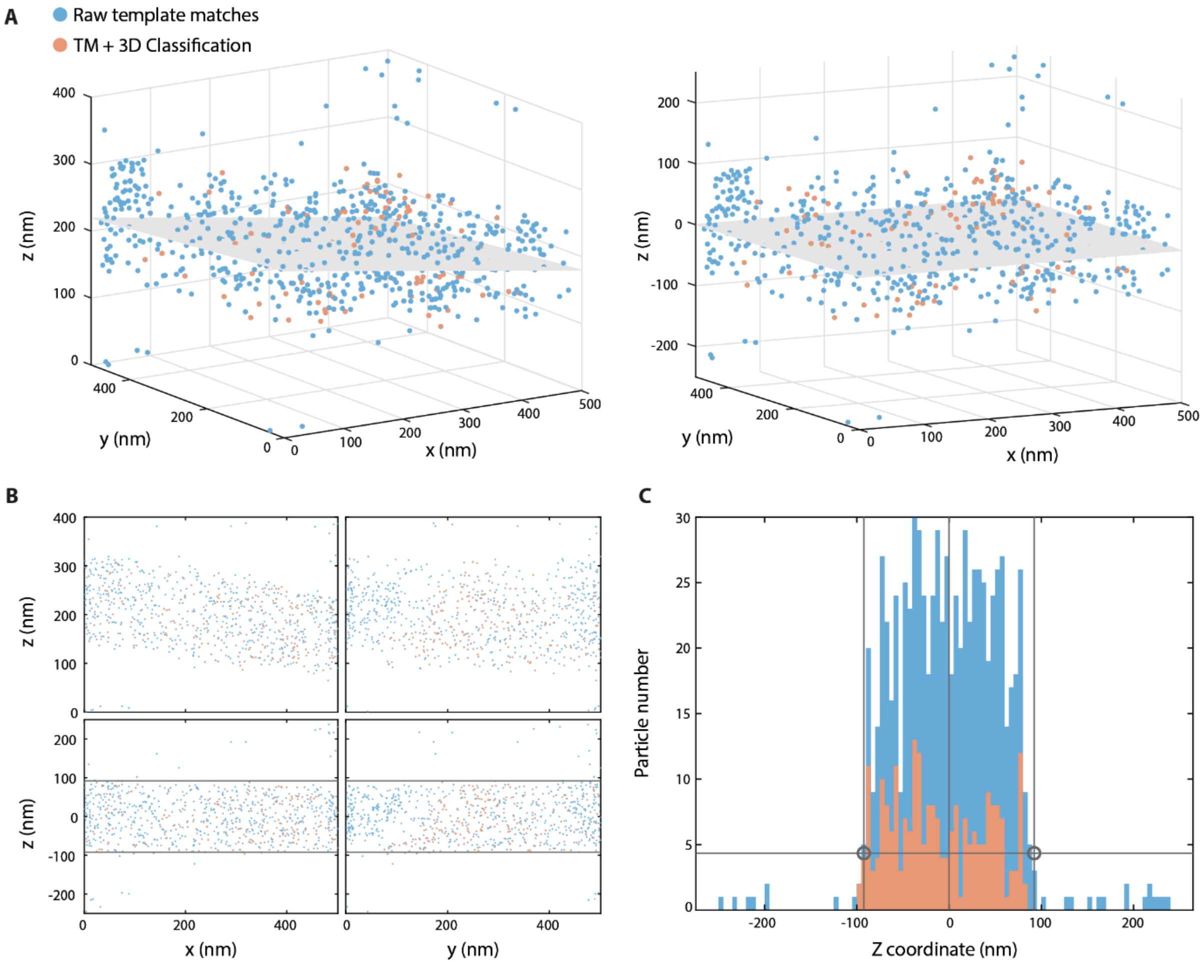

Fig. S7. ***In silico*** **straightening of lamellae and ribosome coordinates to find lamellae edges.** **(A)** Locations of ribosome template matches from a single tomogram are used to fit a 2D plane (left) which is used to straighten the lamella (right). **(B)** X- (left) and Y-(right) ribosome coordinates before (top) and after (bottom) straightening. The grey lines represent the automatically detected edges of the lamella. **(C)** Histogram of straightened z-coordinates of the raw (blue) and classified (red) template matches. Lamella edges were determined based on the drop-off value of the coordinates, indicated with black circles.

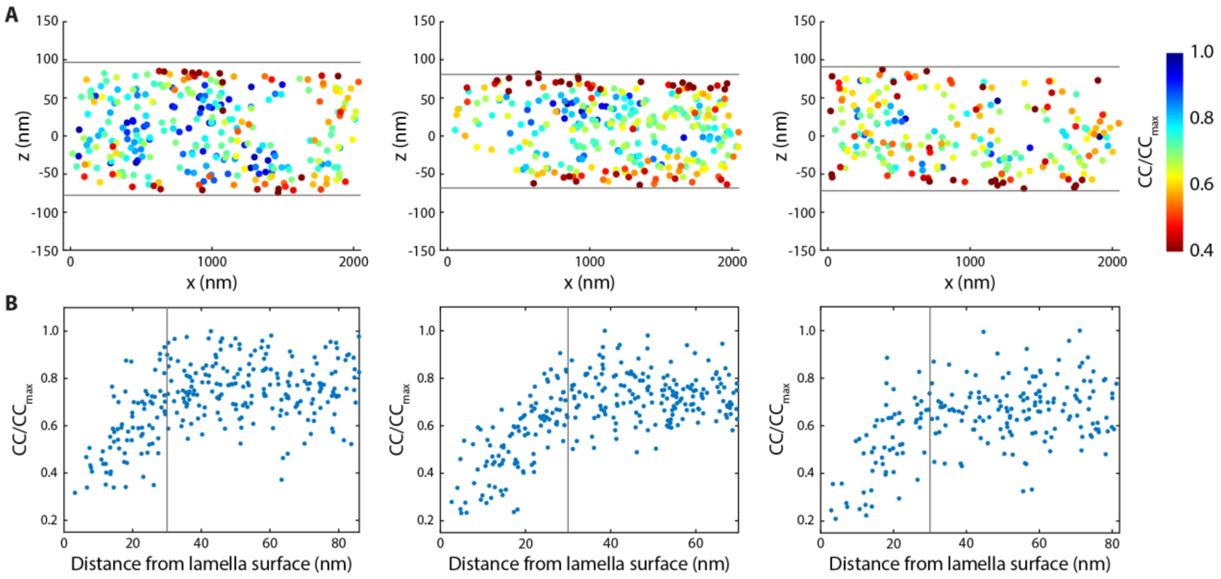

Fig. S8. High-confidence template matching at high resolution. (A) Normalised CC-scores from 3D high-confidence template matching on tomograms with a bin-factor 2 (voxel size 2.4 Å). Lamellae surfaces are displayed as horizontal lines. **(B)** Normalised CC-scores plotted versus the distance from the lamella surface, showing the same tomogram as shown directly above in panel A. Horizontal lines represent 30 nm found as ion-damage zone found using the STA approach.

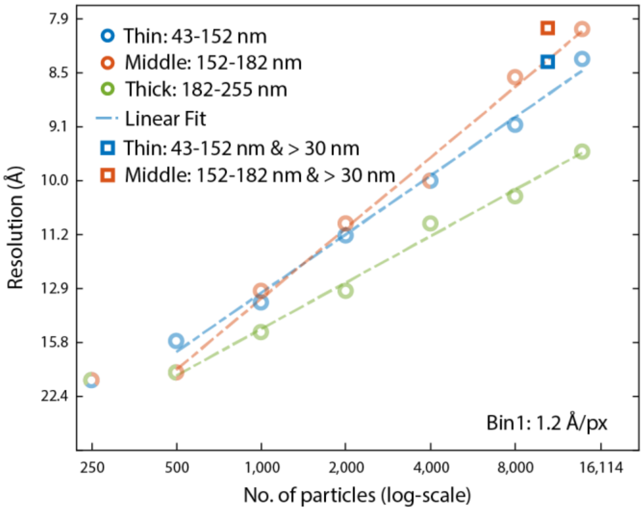

Fig. S9. Resolution plot for lamellae with various local thickness, excluding the ion-damage Particles originating closer than 30 nm from the lamella surface were removed from the thin and middle thickness group, resulting in ca. 10.500 particles. Note that all tomograms showing a local lamella thickness thinner than 60 nm had to be discarded for this analysis. Particles from the middle group still slightly outperformed the particles from the thinner group, so the difference between these groups cannot be explained by the proportionally larger subset of damaged particles from thin parts of the lamellae.

Iteration step	Geometry					CTF	
	Image warp grid	Particle poses	Doming	Stage angles	Volume warp grid	Defocus	Grid search
**1**	3 × 3	✓	-	✓	3 × 3 × 2 × 10	-	-
**2**	6 × 6	✓	✓	✓	6 × 6 × 8 × 10	-	-
**3**	-	-	-	-	-	✓	✓
**4**	7 × 7	✓	✓	✓	7 × 7 × 8 × 10	✓	✓
**5**	10 × 10	✓	✓	✓	10 × 10 × 12 × 10	✓	✓

Table S1. M refinement iterations. Iteration step 3 was only performed when the resolution was at least 7.0 Å. Iteration steps 3-5 were only performed when the resolution still improved after performing the previous iteration step

REFERENCES AND NOTES

1. R. I. Koning, A. J. Koster, T. H. Sharp, Advances in cryo-electron tomography for biology and medicine. *Ann. Anat.* **217**, 82–96 (2018).

2. M. Turk, W. Baumeister, The promise and the challenges of cryo-electron tomography. *FEBS Lett.* **594**, 3243–3261 (2020).

3. P. Zhang, Advances in cryo-electron tomography and subtomogram averaging and classification. *Curr. Opin. Struct. Biol.* **58**, 249–258 (2019).

4. J. A. Briggs, Structural biology in situ—The potential of subtomogram averaging. *Curr. Opin. Struct. Biol.* **23**, 261–267 (2013).

5. F. J. B. Bäuerlein, W. Baumeister, Towards visual proteomics at high resolution. *J. Mol. Biol.* **433**, 167187 (2021).

6. M. Beck, W. Baumeister, Cryo-electron tomography: Can it reveal the molecular sociology of cells in atomic detail? *Trends Cell Biol.* **26**, 825–837 (2016).

7. A. Christoph, A. J. Diebolder, R. I. Koster, Koning, pushing the resolution limits in cryo electron tomography of biological structures. *J. Microsc.* **248**, 1–5 (2012).

8. A. J. Noble, V. P. Dandey, H. Wei, J. Brasch, J. Chase, P. Acharya, Y. Z. Tan, Z. Zhang, L. Y. Kim, G. Scapin, M. Rapp, E. T. Eng, W. J. Rice, A. Cheng, C. J. Negro, L. Shapiro, P. D. Kwong, D. Jeruzalmi, A. des Georges, C. S. Potter, B. Carragher, Routine single particle cryoEM sample and grid characterization by tomography. *eLife* **7**, e34257 (2018).

9. K. Neselu, B. Wang, W. J. Rice, C. S. Potter, B. Carragher, E. Y. D. Chua, Measuring the effects of ice thickness on resolution in single particle cryo-EM. *J. Struct. Biol. X* **7**, 100085 (2023).

10. L. Xue, S. Lenz, M. Zimmermann-Kogadeeva, D. Tegunov, P. Cramer, P. Bork, J. Rappsilber, J. Mahamid, Visualizing translation dynamics at atomic detail inside a bacterial cell. *Nature* **610**, 205–211 (2022).

11. W. J. Rice, A. Cheng, A. J. Noble, E. T. Eng, L. Y. Kim, B. Carragher, C. S. Potter, Routine determination of ice thickness for cryo-EM grids. *J. Struct. Biol.* **204**, 38–44 (2018).

12. B. Turoňová, M. Sikora, C. Schürmann, W. J. H. Hagen, S. Welsch, F. E. C. Blanc, S. von Bülow, M. Gecht, K. Bagola, C. Hörner, G. van Zandbergen, J. Landry, N. T. D. de Azevedo, S. Mosalaganti, A. Schwarz, R. Covino, M. D. Mühlebach, G. Hummer, J. Krijnse Locker, M. Beck, In situ structural analysis of SARS-CoV-2 spike reveals flexibility mediated by three hinges. *Science* **370**, 203–208 (2020).

13. J. Mahamid, S. Pfeffer, M. Schaffer, E. Villa, R. Danev, L. Kuhn Cuellar, F. Förster, A. A. Hyman, J. M. Plitzko, W. Baumeister, Visualizing the molecular sociology at the HeLa cell nuclear periphery. *Science* **351**, 969–972 (2016).

14. C. Berger, N. Premaraj, R. B. G. Ravelli, K. Knoops, C. López-Iglesias, P. J. Peters, Cryo-electron tomography on focused ion beam lamellae transforms structural cell biology. *Nat. Methods*, **20**, 499–511 (2023).

15. M. Marko, C. Hsieh, R. Schalek, J. Frank, C. Mannella, Focused-ion-beam thinning of frozen-hydrated biological specimens for cryo-electron microscopy. *Nat. Methods* **4**, 215–217 (2007).

16. M. Schaffer, B. D. Engel, T. Laugks, J. Mahamid, J. M. Plitzko, W. Baumeister, Cryo-focused ion beam sample preparation for imaging vitreous cells by cryo-electron tomography. *Bio. Protoc.* **5**, e1575 (2015).

17. P. C. Hoffmann, J. P. Kreysing, I. Khusainov, M. W. Tuijtel, S. Welsch, M. Beck, Structures of the eukaryotic ribosome and its translational states in situ. *Nat. Commun.* **13**, 7435 (2022).

18. H. Xing, R. Taniguchi, I. Khusainov, J. P. Kreysing, S. Welsch, B. Turoňová, M. Beck, Translation dynamics in human cells visualized at high resolution reveal cancer drug action. *Science* **381**, 70–75 (2023).

19. C. Berger, M. Dumoux, T. Glen, N. B. Y. Yee, J. M. Mitchels, Z. Patáková, M. C. Darrow, J. H. Naismith, M. Grange, Plasma FIB milling for the determination of structures in situ. *Nat. Commun.* **14**, 629 (2023).

20. C. A. Volkert, A. M. Minor, Focused ion beam microscopy and micromachining. *MRS Bullet.* **32**, 389–399 (2007).

21. L. A. Giannuzzi, F. A. Stevie, *Introduction to Focused Ion Beams: Instrumentation, Theory, Techniques and Practice* (Springer, 2005), vol. 193.

22. L. A. Giannuzzi, F. A. Stevie, A review of focused ion beam milling techniques for TEM specimen preparation. *Micron* **30**, 197–204 (1999).

23. J. Mayer, L. A. Giannuzzi, T. Kamino, J. Michael, TEM sample preparation and FIB-induced damage. *MRS Bullet.* **32**, 400–407 (2007).

24. B. A. Lucas, N. Grigorieff, Quantification of gallium cryo-FIB milling damage in biological lamellae. *Proc. Natl. Acad. Sci. U S A* **120**, e2301852120 (2023).

25. B. A. Lucas, B. A. Himes, L. Xue, T. Grant, J. Mahamid, N. Grigorieff, Locating macromolecular assemblies in cells by 2D template matching with cisTEM. *eLife* **10**, e68946 (2021).

26. B. Turoňová, W. J. H. Hagen, M. Obr, S. Mosalaganti, J. W. Beugelink, C. E. Zimmerli, H.-G. Kräusslich, M. Beck, Benchmarking tomographic acquisition schemes for high-resolution structural biology. *Nat. Commun.* **11**, 876 (2020).

27. J. R. Kremer, D. N. Mastronarde, J. R. McIntosh, Computer visualization of three-dimensional image data using IMOD. *J. Struct. Biol.* **116**, 71–76 (1996).

28. M. Schaffer, J. Mahamid, B. D. Engel, T. Laugks, W. Baumeister, J. M. Plitzko, Optimized cryo-focused ion beam sample preparation aimed at in situ structural studies of membrane proteins. *J. Struct. Biol.* **197**, 73–82 (2017).

29. D. Tegunov, P. Cramer, Real-time cryo-electron microscopy data preprocessing with Warp. *Nat. Methods* **16**, 1146–1152 (2019).

30. W. Wan, S. Khavnekar, J. Wagner, P. Erdmann, W. Baumeister, STOPGAP: A software package for subtomogram averaging and refinement. *Microsc. Microanal.* **26**, 2516–2516 (2020).

31. J. Zivanov, T. Nakane, B. O. Forsberg, D. Kimanius, W. J. Hagen, E. Lindahl, S. H. Scheres, New tools for automated high-resolution cryo-EM structure determination in RELION-3. *eLife* **7**, e42166 (2018).

32. D. N. Mastronarde, S. R. Held, Automated tilt series alignment and tomographic reconstruction in IMOD. *J. Struct. Biol.* **197**, 102–113 (2017).

33. P. B. Rosenthal, R. Henderson, Optimal determination of particle orientation, absolute hand, and contrast loss in single-particle electron cryomicroscopy. *J. Mol. Biol.* **333**, 721–745 (2003).

34. D. Tegunov, L. Xue, C. Dienemann, P. Cramer, J. Mahamid, Multi-particle cryo-EM refinement with M visualizes ribosome-antibiotic complex at 3.5 Å in cells. *Nat. Methods* **18**, 186–193 (2021).

35. S. Cruz-León, T. Majtner, P. C. Hoffmann, J. P. Kreysing, M. W. Tuijtel, S. L. Schaefer, K. Geißler, M. Beck, B. Turoňová, G. Hummer, High-confidence 3D template matching for cryo-electron tomography. bioRxiv 2023.09.05.556310 [Preprint] (2023). https://doi.org/10.1101/2023.09.05.556310.

36. R. D. Kelley, K. Song, B. V. Leer, D. Wall, L. Kwakman, Xe+ FIB milling and measurement of amorphous silicon damage. *Microsc. Microanal.* **19**, 862–863 (2013).

37. Q. Yang, C. Wu, D. Zhu, J. Li, J. Cheng, X. Zhang, The reduction of FIB damage on cryo-lamella by lowering energy of ion beam revealed by a quantitative analysis. *Structure* **31**, 1275–1281.e4 (2023).

38. N. I. Kato, Reducing focused ion beam damage to transmission electron microscopy samples. *J. Electron Microsc. (Tokyo)* **53**, 451–458 (2004).

39. A. Al-Amoudi, D. Studer, J. Dubochet, Cutting artefacts and cutting process in vitreous sections for cryo-electron microscopy. *J. Struct. Biol.* **150**, 109–121 (2005).

40. S. Klumpe, H. K. Fung, S. K. Goetz, I. Zagoriy, B. Hampoelz, X. Zhang, P. S. Erdmann, J. Baumbach, C. W. Müller, M. Beck, J. M. Plitzko, J. Mahamid, A modular platform for automated cryo-FIB workflows. *eLife* **10**, e70506 (2021).

41. W. J. H. Hagen, W. Wan, J. A. G. Briggs, Implementation of a cryo-electron tomography tilt-scheme optimized for high resolution subtomogram averaging. *J. Struct. Biol.* **197**, 191–198 (2017).

42. T. Grant, N. Grigorieff, Measuring the optimal exposure for single particle cryo-EM using a 2.6 Å reconstruction of rotavirus VP6. *eLife* **4**, e06980 (2015).

43. A. Burt, alisterburt/dynamo2m (2020); https://github.com/alisterburt/dynamo2m.

44. S. Chaaban, sami-chaaban/starparser (2023); https://github.com/sami-chaaban/starparser.

45. T. D. Goddard, C. C. Huang, E. C. Meng, E. F. Pettersen, G. S. Couch, J. H. Morris, T. E. Ferrin, UCSF ChimeraX: Meeting modern challenges in visualization and analysis. *Protein Sci.* **27**, 14–25 (2018).

46. J. B. Heymann, The progressive spectral signal-to-noise ratio of cryo-electron micrograph movies as a tool to assess quality and radiation damage. *Comput. Methods Programs Biomed.* **220**, 106799 (2022).
